# A Comparative Study on the Biological Characteristics of Human Adipose-Derived Stem Cells from Lipectomy and Liposuction

**DOI:** 10.1371/journal.pone.0162343

**Published:** 2016-09-09

**Authors:** Yongqian Bian, Chen Deng, Wangzhou Li, Zhanjun Lei, Yuejun Li, Xueyong Li

**Affiliations:** Department of Burn and Plastic Surgery, Tangdu Hospital, Fourth Military Medical University, Xi'an, Shannxi, China; Universita degli Studi di Torino, ITALY

## Abstract

**Purposes:**

To compare the biological behaviors of human adipose-derived stem cells (ADSCs) isolated from adipose tissue by lipectomy and liposuction, with the purpose of providing the basis for clinical application.

**Methods:**

The proliferation and apoptosis of ADSCs were analyzed by CCK-8 assay and flow cytometry. Cell migration was measured by a wound healing assay. An ELISA assay was used to evaluate paracrine functions. SOD and MDA were tested by xanthine oxidase and thiobarbituric acid reactions, respectively. In addition, we used a CCK-8, LDH assay and flow cytometry to analyze the proliferation and apoptosis of ADSCs treated with lidocaine or adrenaline.

**Results:**

The viable ADSCs yield from liposuction was significantly lower than that from lipectomy, while the apoptosis of cells from liposuction was significantly higher than from lipectomy. The paracrine secretion of the two sources of ADSCs was highest when treated with 10^−7^ mol/L insulin and 10 ng/mL TGF-α, but there were no significant differences in VEGF, IL-6, IL-8 or HGF levels. The ADSCs from lipectomy migrated faster than those from liposuction, and SOD in the lipectomy group was higher than in the liposuction group, whereas MDA of the lipectomy group was lower than that of the liposuction group. The proliferation ADSCs treated with lidocaine or adrenaline was greatly decreased, while apoptosis was significantly increased, and cytotoxicity of lidocaine or adrenaline to ADSCs was dose-dependent.

**Conclusions:**

Compared with ADSCs from liposuction, the ADSCs from lipectomy have better biological characteristics. Lidocaine and adrenaline decreased the viability of ADSCs, and their cytotoxicity to ADSCs was dose-dependent.

## Introduction

Since ADSCs were first isolated from lipoaspirates by Zuk in 2001[1], they were gradually found to have the abilities of self-renewal, multiple differentiation potential [2, 3], and paracrine secretion of many important growth factors. Previous research has indicated that ADSCs can notably increase the survival rate of autologous fat transplantation through reducing necrosis, liquefaction, fibrosis, calcification, etc. Therefore, fat grafting has been used in a wide spectrum of clinical applications, such as facial plastic and reconstructive surgery, breast reconstruction and augmentation, and the repair of soft tissue deficiencies resulting from congenital defects, trauma, and tumors [4, 5].

Liposuction and lipectomy are two of the most important ways to obtain ADSCs clinically. However, there is no sufficient evidence to support which type of ADSCs have better biological activity and differentiation potential. Some clinical studies showed that autologous fat transplantation utilizing the adipose tissue from liposuction has a lower survival rate due to partial necrosis, liquefaction, and fibrosis. Local tumescent anesthesia fluid in liposuction is thought to be a crucial factor that has a cytotoxic effect that reduces cell viability. Previous studies have reported that lidocaine can increase the fibrosis and necrosis of fatty grafting tissue, and decrease the cell activity and pluripotent differentiation of stem cells [6–8]. In a study of pluripotent differentiation potential, Schreml et al found that a significantly lower number of cultures obtained from liposuction than from lipectomy could be differentiated into osteocytes and chondrocytes [9].

Currently, there is little systematic and integrated research available on the cytotoxicity of liposuction and lipectomy to ADSCs. Given the wide spectrum clinical applications of ADSCs, we compared the biological characteristics of the cells from liposuction and lipectomy in terms of cell morphology, growth kinetics, and function, with the purpose of learning the most effective way to obtain ADSCs [10]. In addition, we detected the cytotoxicity of local tumescent liposuction to ADSCs through treating the cells with different concentrations of lidocaine and adrenaline, which can provide a reference for the clinical application of ADSCs.

## Materials and Methods

### Ethics statement

This study was approved by the ethics committee of the Fourth Military Medical University (Assigned No. TDLL-2015536) and all experimental procedures were conducted in accordance with ethical guidelines and the Declaration of Helsinki. All patients involved in the study provided their written informed consent for participating.

### Isolation and identification of ADSCs

Between April and July 2015, 10 patients who underwent liposuction and 10 patients who underwent lipectomy in Tangdu Hospital, the Fourth Military Medical University were selected to participate in the study. After patients’ adipose tissues were collected, ADSCs were isolated immediately as described in previous reports [11, 12]. Briefly, adipose tissues were digested with 0.1% collagenase Ι (Gibco) for 1 hour at 37°C with shaking every 15 minutes. Enzymatic dissociation of tissue was stopped by the control medium, and the cell suspension was filtered through a 200 *μ*m mesh (Millipore). The pelleted cells were resuspended in LG-DMEM (Hyclone) supplemented with 10% fetal bovine serum (Sciencell) and 1% antibiotic/antimitotic (Hyclone). The cells were seeded on polystyrene culture flasks and cultured at 37°C in 5% CO_2_. The medium was replaced every 3 days. When reaching 80% confluency, cells were passaged using 0.25% trypsin-EDTA (Millpore).

The isolated human ADSCs at passage 2 were used for surface immunophenotype characterization by cell flow cytometry from BD. The ADSCs were analyzed with a panel of three positive markers (CD73, 90 and 105) and two negative markers (CD34 and 45). The samples without any antibody were set as the negative control group.

### Cell morphology and viability assay

A cell suspension was mixed with 0.4% trypan blue, and the numbers of viable and dead cells were counted by light microscopy. Cellular viability was calculated according to the formula: living cell rate (%) = total living cellular score/ (total living cellular score + total dead cellular score) × 100%.

ADSCs were seeded in 96-well plates in triplicate at 1×10^4^ cells/well. Every 2 hours, cells in the culture supernatant were harvested and counted. Cellular adhesion rate was calculated according to the formula: adhesion rate (%) = (total plated cellular score (1×10^4^)–total supernatant cellular score)/ total plated cellular score × 100%.

### Cell proliferation kinetics assay

Cell proliferation of ADSCs at passage 2 were detected by a WST-8 (2-(2-methoxy-4-nitrophenyl)-3-(4-nitrophenyl)-5-(2,4-disulfophenyl)-2H-tetrazolium) assay. Cells were seeded in 96-well plates at 2×10^3^ cells/well, and cultured with 10% cell counting kit-8 (CCK-8) for 4 hours at 37°C. The absorbance of each well was evaluated spectrophotometrically at 450 nm. Proliferation rates were determined daily for 10 days. These experiments were carried out in quadruplicate.

Apoptosis of ADSCs at passage 2 were assessed by flow cytometry with Annexin V-FITC/PI double staining. Cells were suspended in binding buffer and then Annexin V-FITC and propidium iodide (PI) were added according to the manufacturer’s instructions (KeyGen Biotech, Nanjin, China). The samples were then analyzed by FACS from BD (4A Biotech, Beijing, China).

### Cell function assay

Cellular migration was measured by a wound healing assay. ADSCs at passage 2 were seeded in 24-well plates, and linear scratch wounds were created with a 200 μL pipette tip when cells reached confluency. Then, cells were maintained in serum-free medium. Images were taken at 0, 6, 12, 24, and 48 hours to visualize migrated cells. The cell migration gap was analyzed by Image J software, and a total of ten areas were selected randomly from each well.

Cellular paracrine ability was measured by enzyme-linked immunosorbent assay (ELISA). ADSCs were seeded in 12-well plates at 5×10^4^ cells/well. When they reached 80% confluency, the media was replaced with LG-DMEM supplemented with 1×10^−8^, 1×10^−7^, or 1×10^−6^ mol/L insulin. After cells incubated for 3 days, the media was collected with 1000 × g centrifugation at 4°C for 15 minutes. VEGF (Vascular Endothelial Growth Factor) and HGF (Hepatocyte Growth Factor) levels were detected by ELISA according to the manufacturer’s instructions. Meanwhile, another assessment of the ADSCs was conducted. When cells reached 80% confluency, the media was replaced with LG-DMEM supplemented with 0.1, 0.5, 2, 5, or 10 ng/ml TNF-α. After cells were incubated for 3 days, the media was collected with 1000 × g centrifugation at 4°C for 15 minutes. IL-6 (interleukin-6) and IL-8 levels were assayed by ELISA according to the manufacturer’s instructions (Huamei, Wuhan, China).

Oxidative stress assessment was evaluated by detection of the superoxide dismutase (SOD) and malondialdehyde (MDA) level through the Xanthine oxidase and thiobarbituric acid methods, respectively. Cell pellets were treated with SOD assay reagents, and then incubated at 37°C for 40 minutes to detect SOD activity. SOD activity was determined by photometric measurement (OD 550 nm) and calculated with the following equation:

$$SOD(U/ml)=\frac{A_{sample}-A_{blank}}{A_{control}}÷50\%\times \frac{3.4\;(Reaction\;volume)}{0.1\;(Sample\;volume)}$$

Meanwhile, cell pellets were treated with MDA assay regents, and then were put in a water bath at 95°C for 15 minutes. The supernatant was collected to detect MDA level by the photometric measurement (OD 532 nm) and calculated by the following equation:

$$MDA(\mu mol/ml)=\frac{A_{sample}-A_{sample\;blank}}{A_{standard}-A_{standard\;blank}}\times standard\;concentration(10\;nmol/L)$$

Adipogenic differentiation was performed according to the previous study [13]. Briefly, cells were stimulated for 2 days in the differentiation medium: LG-DMEM supplemented with 10% FBS, 1 μM Dexamethasone, 0.5 mM 3-isobutyl-1-methylxanthine (IBMX), and 10 μg/ml Insulin. Afterwards, cell were cultured for another 2 days with 10 μg/ml insulin alone in LG-DMEM containing 10% FBS. The medium was replaced every other day. After 7 days culture, cells were fixed with 4% formaldehyde, and then stained with a 60% Oil Red O solution. For quantitative analysis, the intracellularly absorbed Oil Red O was extracted in 100% isopropanol, and absorbance was measured at 510 nm by spectrophotometry. All experiments were performed in triplicate.

### Interventions of tumescence liquid

ADSCs derived from lipectomy at passage 2 were seeded in 96-well plates at 2×10^3^ cells/well. After 1 day of culture, ADSCs were treated with 0.5 g/L, 1 g/L, or 2 g/L lidocaine or 0.5 mg/L, 1 mg/L, or 2 mg/L adrenaline, respectively. Cells cultured without lidocaine or adrenaline were used as controls. A CCK-8 assay was used to detect the growth of ADSCs.

Beginning 2 hours after ADSCs were treated with lidocaine or adrenaline, apoptosis rates were detected every day for 10 days. Meanwhile, the supernatant was collected and treated with the LDH (lactate dehydrogenase) assay reagents, and the LDH activity was determined by the photometric measurement of OD at 532 nm.

### Statistical analysis

All data were expressed as the mean ± SD. Statistical evaluation of the results was performed by analysis of variance (ANOVA). Differences identified by ANOVA were pinpointed by unpaired Student’s t-tests. A value of p < 0.05 was considered statistically significant.

## Results

### Cell characterization and viability

**Fig 1:**
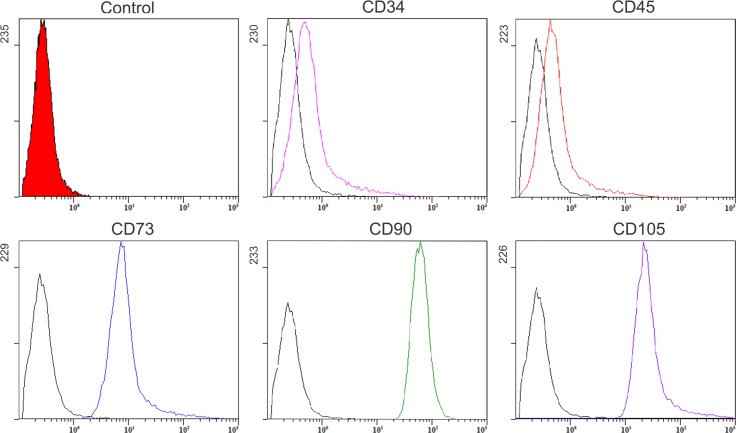
Identification of ADSCs (positive markers: CD73, CD90 and CD105; negative marker: CD34 and CD45) by flow cytometry analysis.

**Fig 2:**
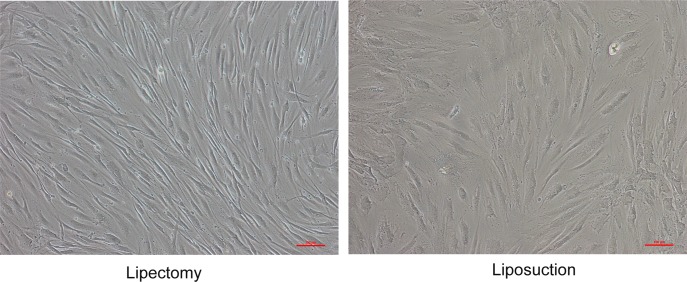
The morphology of ADSCs at passage 3. (A) ADSCs isolated from lipectomy, (B) ADSCs isolated from liposuction.

In order to characterize the cell populations, representative surface marker expression profiles for ADSCs at passage 2 were analyzed. Flow cytometry assay showed that ADSCs expressed the mesenchymal positive markers CD73, CD90 and CD105, but were negative for CD34 and CD45 (Fig 1). The morphology of ADSCs from lipectomy and liposuction were spindle-like. However, the ADSCs at passage 3 from lipectomy arranged in order, whereas the morphology of some ADSCs from liposuction was irregular and the directionality of those cells was worse than that of ADSCs from lipectomy (Fig 2).

**Fig 3:**
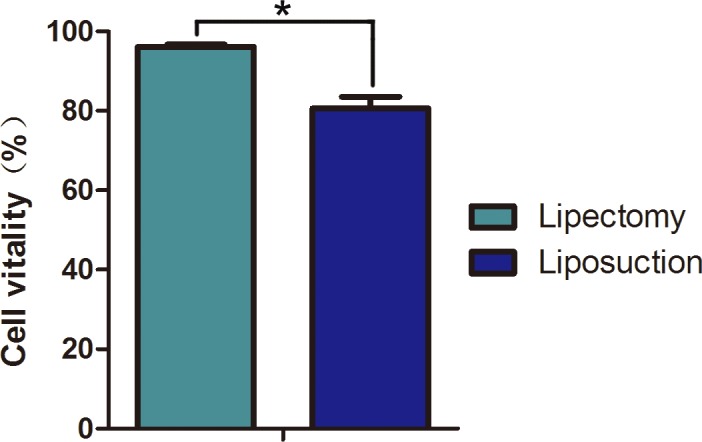
Cell viability of ADSCs from lipectomy and liposuction detected by Trypan blue staining (n = 3, * p<0.05).

**Fig 4:**
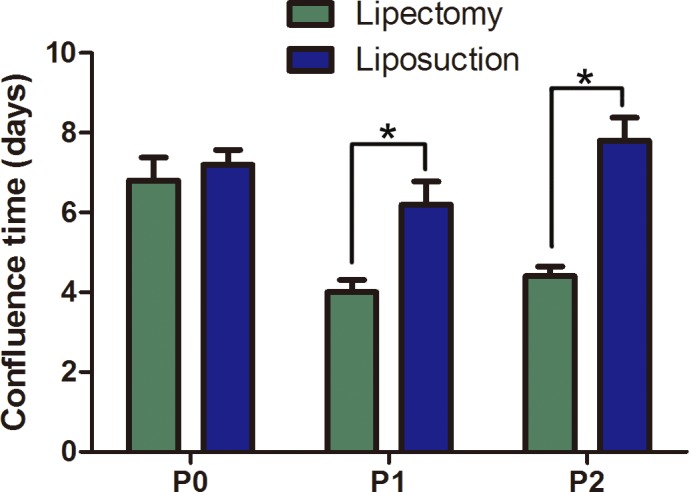
Cell confluence time of ADSCs from lipectomy and liposuction (n = 3,* p<0.05).

**Fig 5:**
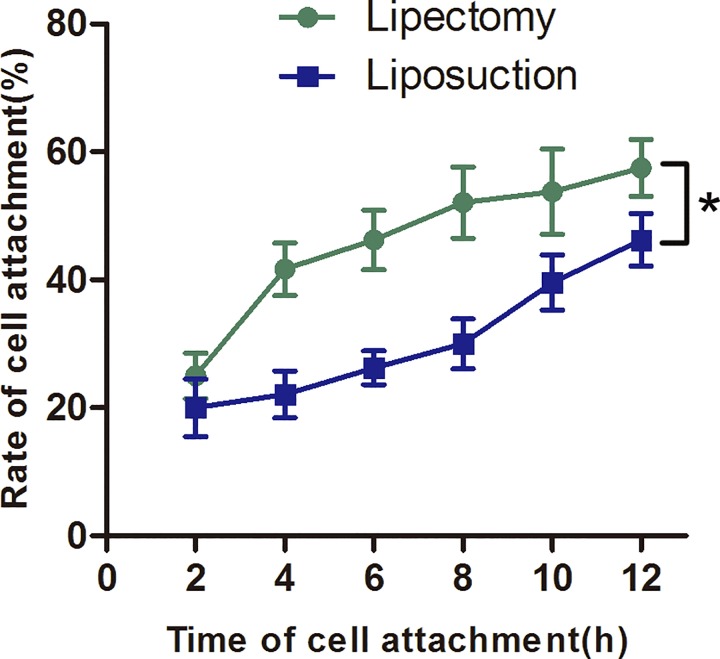
Cell attachment rate of ADSCs from lipectomy and liposuction (n = 3, * p<0.05).

Next, cellular survival rate and confluence time were tested to assay the viability of ADSCs. Trypan blue staining indicated that ADSCs at passage 2 from lipectomy had a higher survival rate than those from liposuction, and the cell viability of the two populations of cells was 96.12±0.60% and 80.59±2.99%, respectively (n = 6, p<0.05) (Fig 3). Because cellular confluence time is an important indicator of cell state, we tested the confluence time of the two populations of cells. The times for ADSCs from lipectomy at primary, passage 1 and passage 2 to reach 80% confluence were 6.80±1.30, 4.00±0.70, and 4.40±0.55 days, respectively, which were significantly shorter than those from liposuction (7.20±0.84, 6.20±1.30, and 7.80±1.30 days, respectively) (n = 5, p<0.05) (Fig 4). Cell attachment rate is another important indicator of cell state. We found that the cell attachment rate of ADSCs at passage 2 from lipectomy was higher than that from liposuction (Fig 5).

### Cell growth kinetics

**Fig 6:**
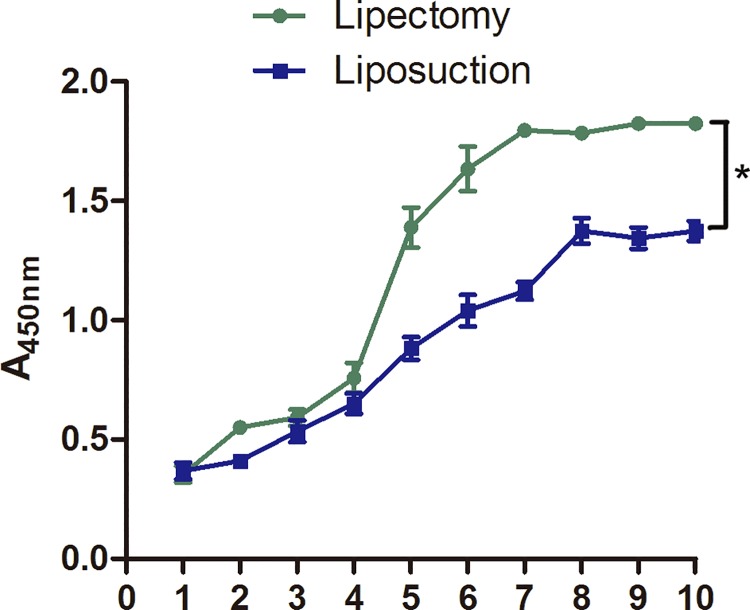
The proliferation of ADSCs from lipectomy and liposuction detected by CCK-8 assay (n = 4, * p<0.05).

A CCK-8 assay indicated that both of the ADSCs showed growth arrest after seeded within 24 hours, a rapid growth phase for 3 to 4 days, and platform phase in 6 days. The proliferation rate of ADSCs from liposuction was lower than that of ADSCs from lipectomy, and accordingly, the platform phase was longer for ADSCs from liposuction than from lipectomy (Fig 6).

**Fig 7:**
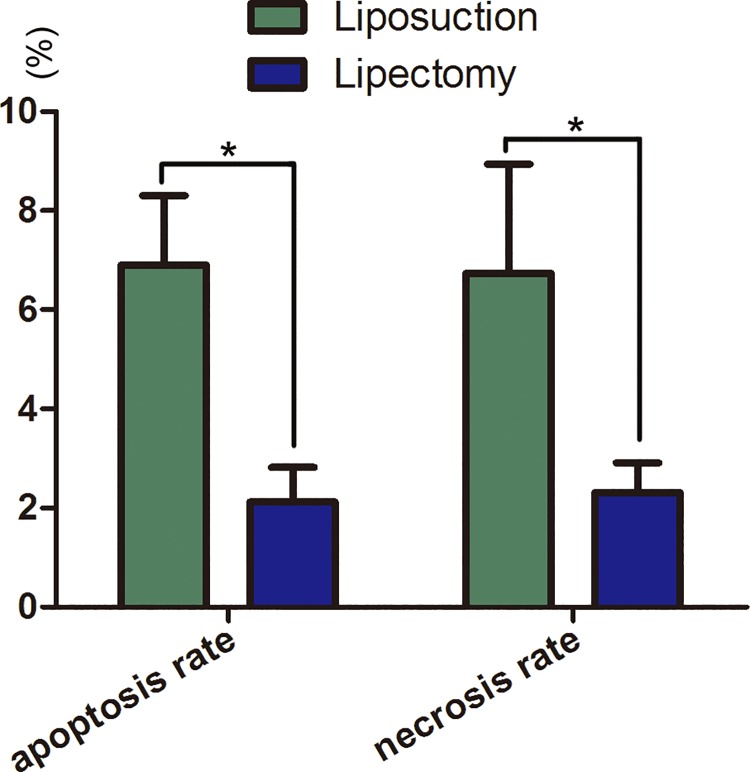
The apoptosis and necrosis of ADSCs from lipectomy and liposuction detected by flow cytometry analysis (n = 3, * p<0.05).

A Flow cytometry assay showed that the apoptosis and necrosis rates of ADSCs at passage 2 from liposuction were 6.90±1.40% and 6.74±2.2%, respectively, both of which were higher than for ADSCs from lipectomy (2.12±0.70% and 2.31±0.60%, respectively) (Fig 7).

### Cell function assay

#### Paracrine secretion assay

**Fig 8:**
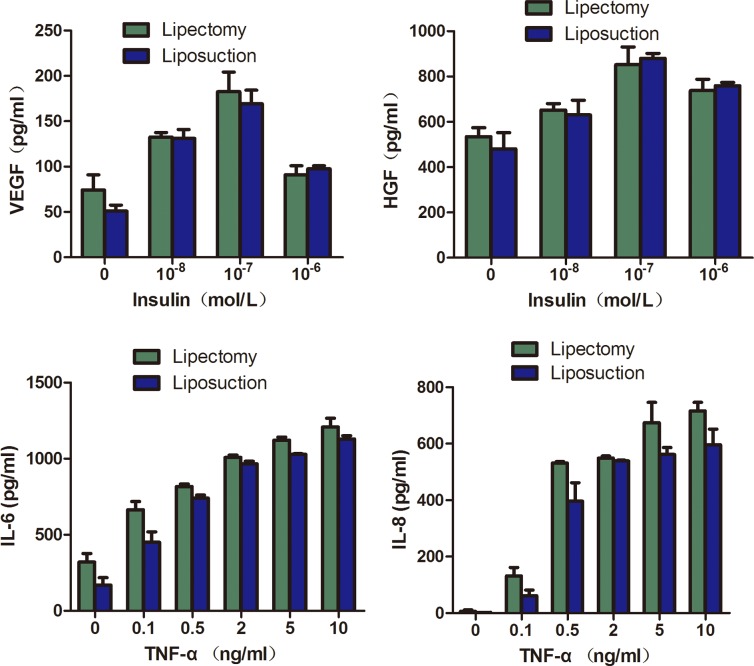
The paracrine secretion of ADSCs measured by ELISA. VEGF (A), IL-6 (B), IL-8 (C), and HGF (D) expression, when ADSCs were treated with different concertations of insulin and TGF-α(n = 3, p>0.05).

Different concentrations of insulin or TGF-α were applied to test the paracrine secretion of ADSCs. We found that ADSCs from both sources had the highest paracrine secretion levels when the concentrations of insulin and TGF-α were 10^−7^ mol/L and 10 ng/ml, respectively. VEGF, IL-6, and IL-8 secreted by ADSCs from lipectomy were (182.67±30.64), (1207.87±57.15), and (716.50±30.00) pg/ml, respectively, which were each higher than the levels secreted by ADSCs from liposuction ((169.33±21.21), (1129.29±21.43), and (596.50±55.00) pg/ml, respectively). Meanwhile, HGF secreted by ADSCs from lipectomy was (852.15±78.57) pg/ml, which was lower than HGF secreted by ADSCs from liposuction ((880±21.43) pg/ml). However, all the data above had no statistically significant differences, which suggest that the two different obtaining methods did not affect the paracrine secretion of ADSCs (Fig 8).

#### Cell migration assay

**Fig 9:**
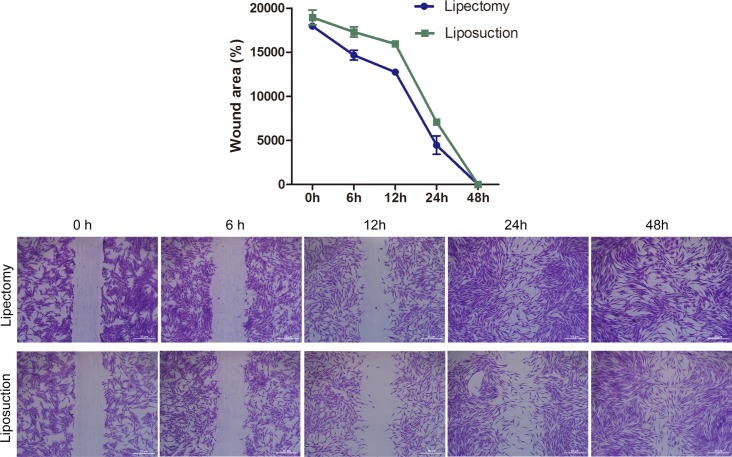
Cell migration of ADSCs as determined by wound scratch assays. (A) Quantification of the “wound gap” distance between the front lines of migrating cells. (B) Images taken at 0, 6, 12, 24, and 48 hours to visualize the wound gap (n = 3, * p<0.05).

A wound scratch assay was used to test cell migration. We found that the scratch area will heal in 48 hours for ADSCs from both sources, but the scratch gap was smaller at 12 and 24 hours for the ADSCs from lipectomy than from liposuction (Fig 9), which indicates that the migration was faster for ADSCs from lipectomy than from liposuction.

#### Oxidative stress assessment

**Fig 10:**
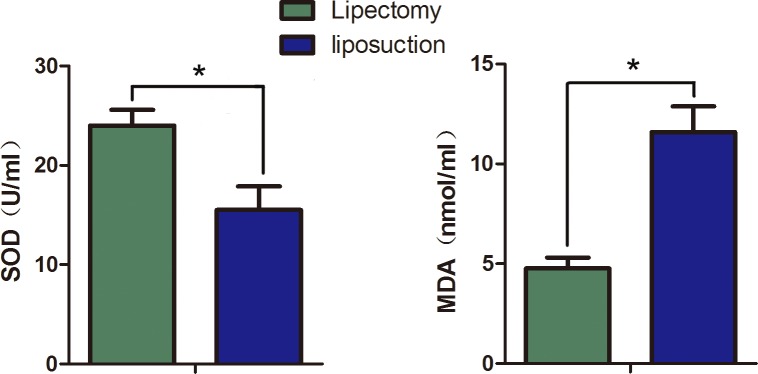
Oxidative stress assessment of ADSCs by detecting the SOD and MDA level. The SOD and MDA levels were detected by Xanthine oxidase and thiobarbituric acid methods, respectively (n = 3, * p<0.05).

Xanthine oxidase and thiobarbituric acid methods were used to detect the SOD and MDA of ADSCs. We found that the SOD of ADSCs from liposuction was 24.00±1.57 U/ml, which was lower than that from lipectomy (15.51±2.37 U/ml); while the MDA of ADSCs from liposuction was 11.59±2.24 nmol/ml, which was higher than that from lipectomy (4.77±0.914 nmol/ml) (Fig 10). These data illustrate that the free radical injury degree was lower for ADSCs from lipectomy than from liposuction. In other words, the ADSCs from lipectomy had a more outstanding capability of scavenging free radicals.

#### Adipogenic differentiation

**Fig 11:**
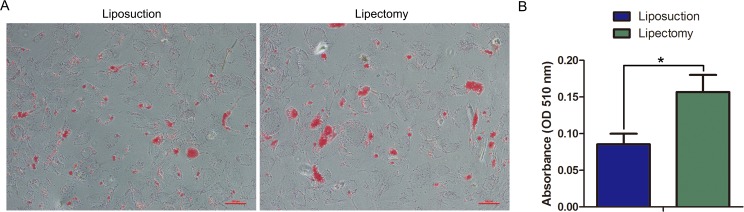
Adipogenic differentiation of ADSCs. (A) Adipose differentiation of ADSCs evaluated by Oil red O staining. (B) Quantitative analysis of the intracellular Oil red O by spectrophotometry (n = 3, * p<0.05).

The adipogenic differentiation of ADSCs from lipectomy and liposuction was assessed at day 7 using Oil red O staining. As shown in Fig 11A, lipid droplets could be detected by Oil red O staining in ADSCs from both sources. However, lipid droplets in the ADSCs from lipectomy were more than that in the cells from liposuction (Fig 11B). Our results illustrated that ADSCs from lipectomy had a better potential of adipogenic differentiation than that from liposuction.

### Tumescent anesthesia fluid intervention

**Fig 12:**
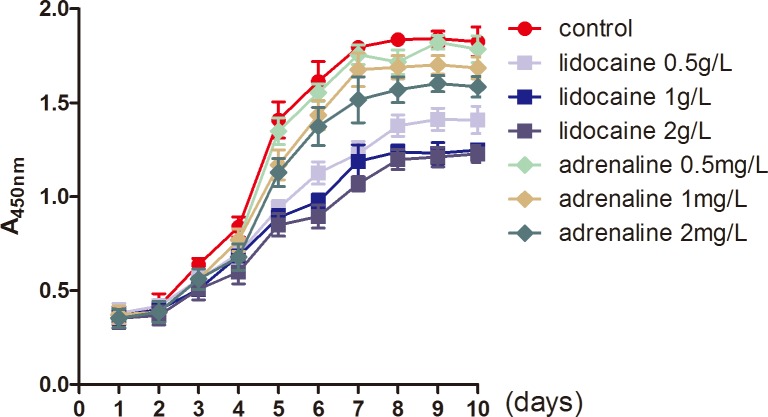
The proliferation of ADSCs, treated with different concertation of lidocaine and adrenaline, detected by CCK-8 assays.

**Fig 13:**
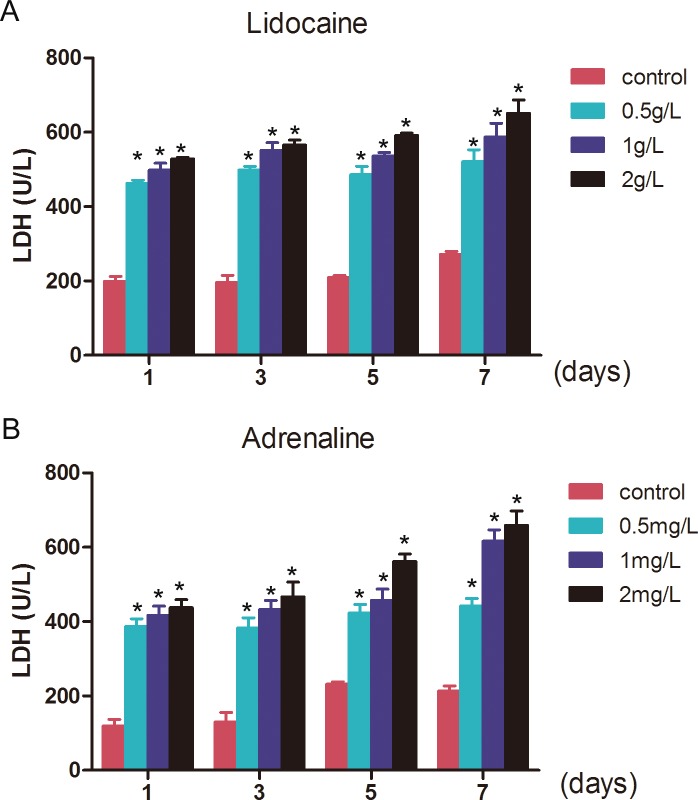
**The LDH levels of ADSCs treated with different concertations of lidocaine (A) and adrenaline (B)** (n = 3, * p<0.05).

**Fig 14:**
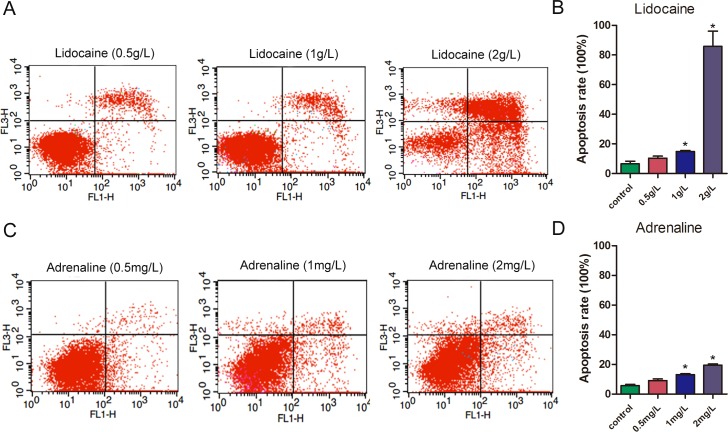
The apoptosis of ADSCs treated with lidocaine or adrenaline. ADSCs were treated with different concentrations of lidocaine (A) or adrenaline (C) for 2 hours, and then the apoptosis of cell was measured using flow cytometry analysis after staining with Annexin V-FITC/PI. (B), (D) Quantification of apoptosis in the cells treated with different concentrations of lidocaine or adrenaline, respectively (n = 3, * p<0.05).

As lidocaine and adrenaline are two of the primary components of the tumescent anesthesia fluid in liposuction, we wondered if their toxicity accounts for the differences in the biological characteristics of ADSCs from lipectomy and liposuction. CCK-8 assays showed that the proliferation of ADSCs was reduced when treated with lidocaine or adrenaline, and the proliferation of ADSCs treated with lidocaine was lower than those treated with adrenaline. Furthermore, the decrease in proliferation of ADSCs treated with lidocaine or adrenaline was dose-dependent (Fig 12). After that, we treated ADSCs with different concentrations of lidocaine or adrenaline and found the LDH level was obviously elevated, which suggests that the structure of the cell membrane was destroyed (Fig 13). Lastly, 2 hours after being treated with different concentrations of lidocaine or adrenaline we found that the apoptosis of the ADSCs was significantly increased and that there was a statistically significant difference between the cells treated with 1 or 2 g/L lidocaine or 1 or 2 mg/L adrenaline (*p*<0.05) (Fig 14).

## Discussions

In recent years, the repair of soft tissue defects resulting from congenital defects, trauma, and tumor is one of the biggest challenges facing plastic surgeons [14]. Previous studies indicate that ADSCs co-transplanted with stromal vascular fraction can increase the survival rate of adipocytes [15]. Further studies have shown that in secondary mammary reconstruction, fat grafting that includes stem cells can achieve better reconstructive outcomes [16]. Considering the significance of ADSCs in repairing soft tissue defects and their application in tissue engineering, it is important to obtain ADSCs with better biological activity and differentiation potential for the sake of current scientific research and later clinical application.

In our research, we obtained ADSCs from lipectomy and liposuction and compared their biological characteristics. We found that ADSCs from both sources were spindle-like, and the morphology changed to an irregular shape with cell passaging. However, there were some cell aging situations in the ADSCs from liposuction, such as the cell body and the nucleolus becoming larger and the nucleus becoming flat. In addition, the proliferation and the anti-oxidative stress capability of ADSCs from liposuction were obviously decreased compared with those from lipectomy. The decline of anti-oxidative stress ability may directly damage the capacity of ADSCs to provide antioxidants and to eliminate toxic substances in the local environment, which may result in poor growth status and even cell death [17]. The paracrine secretion of ADSCs can promote angiogenesis and regulate the tissue microenvironment by secreting VEGF, IL-6, IL-8, HGF, EGF, bFGF, TGF-β, etc. [18, 19]. In our research, we found ADSCs from lipectomy can secrete more VEGF, IL-6, and IL-8 and less HGF than those from liposuction, but the difference was not statistically significant, suggesting that liposuction may not affect the paracrine secretion of ADSCs.

Previous studies have suggested that the tumescent anesthesia fluid may account for the decline of the proliferation and anti-oxidants of ADSCs [20, 21]. As lidocaine and adrenaline are two of the primary components of the tumescent anesthesia fluid used in liposuction [22], we treated the ADSCs with lidocaine or adrenaline with respect to the clinical doses, and then tested the cell proliferation, apoptosis, and LDH levels. We found that the proliferation was obviously inhibited and the apoptosis and necrosis were increased when ADSCs were treated with lidocaine or adrenaline. Our results corroborate those found by prior research. Li found that lidocaine can promote endoplasmic reticulum stress in SH-SY5Y cells and active caspase 3, resulting in cell apoptosis [23]. Chang indicated that in thyroid cancer, lidocaine caused disruption of the mitochondrial membrane, attenuated ERK1/2 activity, and induced activation of MAPK and c-jun N-terminal kinase, which led to cell apoptosis [24]. Similarly, lidocaine can also induce apoptosis in neuroblastoma and neuronal cell lines [25, 26]. As a type of neurotransmitter and hormone *in vivo*, adrenaline plays an important role in physiological regulation. However, high concentrations of adrenaline can damage the DNA via ROS in lymphocytes [27]. Research on the cytotoxicity of lidocaine and adrenaline to ADSCs is relatively scarce, but some researchers have found that a minimum of three rinses of graft adipose tissue achieved a substantial reduction of the lidocaine concentrations, which led to satisfactory effects on the transplantation of viable adipocytes and precursor cells for contour augmentation, enlargement, or filling of defects [28]. Taken together, the tumescent anesthesia fluid, which includes lidocaine and adrenaline, used in liposuction can inhibit cell viability and functioning.

## Conclusions

Our study compared the biological behaviors of ADSCs isolated from fat tissue by lipectomy and liposuction in terms of cell viability, proliferation, migration, paracrine secretion, and anti-oxidative stress capability, and the results indicate that ADSCs from lipectomy have better biological characteristics. Moreover, lidocaine and adrenaline, which are primary components of the tumescent anesthesia fluid used in liposuction, decreased the viability of ADSCs, and their cytotoxicity to ADSCs was dose-dependent. In all, these data may provide an important reference not only for current scientific research but also for the clinical application of ADSCs.
